# The Relationship Between Preschool Inclusive Education Teachers’ Organizational Support and Work Engagement: The Mediating Role of Teacher Self-Efficacy

**DOI:** 10.3389/fpsyg.2022.900835

**Published:** 2022-05-24

**Authors:** Chunlan Jiao, Jing Qian, Huan Liu

**Affiliations:** Normal School, Changshu Institute of Technology, Suzhou, China

**Keywords:** preschool inclusive education, organizational support, teacher self-efficacy, work engagement, mediation effect

## Abstract

This study aims to explore the relationship and mechanism between the preschool inclusive education teachers’ organizational support, teacher self-efficacy, and work engagement. This study adopted the organizational support scale, inclusive education efficacy scale, and work engagement scale, measured for 600 preschool inclusive education teachers, eventually obtained 568 effective questionnaires, established research model, and analyzed the data using the structural equation model (SEM). There are significantly more men (65.1%) than women (34.9), and the majority (57.6%) were public kindergarten. Organizational support significantly positively affects teachers’ self-efficacy (β = 0.526, *p* < 0.001) and work engagement (β = 0.385, *p* < 0.001) in preschool inclusive education. Preschool teachers’ self-efficacy has a significant positive impact on work engagement (β = 0.222, *p* < 0.001). Preschool teachers’ self-efficacy plays a partial mediating role between organizational support and work engagement (β = 0.202, *p* < 0.001, CIs = [0.077, 0.305]). Organizational support not only directly affects teachers’ self-efficacy and work engagement in preschool inclusive education but also indirectly affects their work engagement through preschool teachers’ self-efficacy, which provides theoretical and practical guidance for the research of inclusive education.

## Introduction

Inclusive education has gradually become the most enthusiastic topics discussed in special education in the world since the 1970s. Inclusion means being fully accepted, which is based on the belief that meets all the needs of all students, educating all children in the educational environment for children age characteristics in ordinary schools. Preschool inclusive education is to let the children who have a special education need to enter ordinary kindergartens and jointly accept conservation and education with ordinary children. The implementation of inclusive education is not only beneficial to the development of special children’s cognition, emotion, sociality, and behavioral skills. It also helps normal children’s compassion and responsibility, self-confidence and mature, self-esteem, self-respect, and social aspects of cultivation (Terpstra and Tamura, 2008). Compared with developed countries, the development of Chinese preschool inclusive education has been fully implemented but is still relatively backward development. At present, China’s inclusive education mainly has many problems with high education, poor working, poor education, and improving related support systems. This hinders high-quality development of Chinese preschool integration education varying degrees.

Pre-preschool education teachers are the subject of implementing inclusive education, and its working conditions will directly affect the quality of integration education and the future development of young children. Many previous studies explore the objective problems of relatively steady states such as preschool teachers with not high inclusive education, lack of professional knowledge and skill, and less concerned about the relative dynamic indicators of preschool teachers’ work participation. Work engagement refers to the mental relationship between individuals and tasks and is a positive and enriched mental state associated with work (Sakuraya et al., 2020). How to improve the working engagement of education for preschool inclusive education teachers? Organizational support provides a new idea for the research of the work engagement of preschool inclusive education teachers. Eisenberger et al. (1986) earlier pointed out that organizational support will enhance the expectations of individuals’ work results and the degree of emotional attachment to the organization, thus paying more efforts to achieve organizational goals. Based on the principle of reciprocity, when employees feel the organization’s support, as an exchange, they will also assume the expectations of the organization to help organizations achieve their goals, thus increasing work engagement. Resource Preservation Theory believes that individuals usually need to rely on various resources to maintain status and growth, so individuals do not only need to use existing internal resources and need to access the external resources required (Eisenberger et al., 1986). Although many studies have confirmed that organizational support will have an impact on work engagement, the research of self-efficacy mediating role is still less. Teachers’ self-efficacy has an important impact on teachers’ work engagement, which affects not only preschool students’ physical and mental development and academic achievements but also teachers’ own work. Whether the preschool teacher is full of vitality in the work, whether to actively dedicate your own time energy to help students grow, and whether you will invest more energy than other teachers to improve themselves, these will be affected by teachers’ self-efficacy. As a kind of school organization, kindergarten has its own uniqueness, which determines the results of other fields that cannot directly help us answer questions in the preschool education. Therefore, this study uses the Resource Preservation Theory as a view of the preschool inclusive education teacher, exploring the impact mechanism of organizational support and self-efficacy on work engagement preschool. This study aims to provide a reference for further promoting the work engagement state of preschool integration education teachers and propose targeted and feasible recommendations for improving the quality and stability of preschool teachers.

This study has the following contributions. First, previous studies have focused on the research on the definition, dimensional division, influencing factors, and the relationship with the variables of employee performance but less research on the work of preschool inclusive education teachers. This study from the perspective of organizational support explored the relationship between the organizational support, self-efficacy, and work engagement with the preschool inclusive education teachers. The study will enrich the theoretical research on organizational support, self-efficacy and work engagement. Second, expand the application of social exchange theory in the field of preschool teachers’ inclusive education. Third, this study can promote kindergarten to provide incentive mechanisms in promoting the work of preschool inclusive education teachers and provides an empirical reference for preschool inclusive education teaching.

One of the purposes of this study is to investigate the relationships between the organizational support, the inclusive education teachers’ self-efficacy, and work engagement by surveying the kindergarten teacher of China. Hence, it is proposed that the inclusive education teachers’ self-efficacy mediates the relationship between organizational support and work engagement. The findings of this study are expected to extend inclusive education literature and provide practical implications for the development of inclusive education.

This study consists of five sections. The “Introduction” section is followed by the theoretical background of this study presented in the “Literature Review and Hypothesis Development” section, while the context of the “Research Methodology” section is the research framework and hypotheses development. Then, the research methods and data analysis are analyzed in detail in the “Data Analysis and Results” section. In the final section, the findings and highlights of the theoretical and practical implications for researchers and companies will be discussed, along with some suggestions for future research according to the limitations of this study.

## Literature Review and Hypothesis Development

### Literature Review

#### Social Exchange Theory

The representative of social exchange theory is Homans (1958), who proposed the behaviorist exchange theory from the perspective of economics and behavioral psychology. He believed that the social relationship between people is based on exchange, and all behaviors are exchange behaviors. The essence of interpersonal relationship is the attribute of social exchange, including material (i.e., time, financial, and physical strength, etc.) and spiritual exchange (i.e., spiritual reward, comfort and enjoyment, social status, identity, and fame, etc.). The core principle is “mutual benefit.”

Social exchange has been applied to the relationship between organizations and employees, which can explain the behavior and motivation of employees and organizations. In the work scene, when the organization gives more support to the employees, the employees expect to get the rewards from the organization with more input, better performance, and loyal attitude. Such mutually beneficial behavior not only increases and upgrades the resources of each other but also strengthens the exchange relationship between the employees and the organization. The results show that there is a close relationship between social exchange and employee contribution. High-quality social exchange can lead to employees’ loyalty commitment to the organization, improve organizational civilized behavior, and increase work engagement (Zhu, 2012). Based on social exchange theory, the relationship between employees and organizations is regarded as a kind of exchange relationship. Based on this exchange ideology, the increase of employees’ engagement in work comes from the expectation that efforts will lead to better results and the emotional connection with the organization. This ideology of exchange stems from the principle of reciprocity, which states that people should help those who have helped them. Organizational support behavior will make employees have a sense of obligation, and they will actively invest time and energy for the organization to help the organization achieve goals and better development. In other words, the existence of organizational support helps employees and organizations to connect a bridge of exchange relationship.

This study takes kindergarten and preschool teachers as the main focus and takes the influencing mechanism of job engagement as the research objective. Since this study takes “social exchange theory” as the fundamental theoretical basis, organizational support is taken as the starting perspective. At the same time, this study is influenced by the current trend of positive organizational behavior theory, hoping to make an in-depth analysis of the influence mechanism between organizational support and job performance from the psychological positive aspect of the research object. Therefore, self-efficacy, a typical category of positive organizational behavior, is introduced as the mediating variable of the theoretical model, and how the mediating effect of self-efficacy is reflected between the independent variable perceived organizational support and the dependent variable job engagement is studied, so as to make the theoretical model of this study more substantial and persuasive.

#### Perceived Organizational Support

The organizational support theory (OST) refers to the emphasis on the organization’s contribution and wellbeing. Social exchange theory believes that higher organizational support enhances employee expectations and organizes emotional links, guiding employees to help achieve organizational goals (Eisenberger et al., 1986). On the basis of Eisenberger research, other scholars have made different opinions on the meaning of organizational support. McMillin (1997) believes that organizations should provide substances, materials, training, and other tool-based support for employees while giving employee emotional support and respect support. The lack of one will weaken other two supports.

Previous studies on the results of employee organization support mainly focused on work performance, organizational commitment, work engagement, and separation intentions. Wayne clearly stated that there is a positive correlation between organizational support and work satisfaction and organization commitment (Wayne et al., 2003). Eisenberger et al. (1986) proves that work engagement is not only positively related to organizational support but to improve employee’s remaining willingness. Kurtessis et al. (2017) meta-analysis of organizational support confirmed that organizational support has a positive impact on work performance. Aselage and Eisenberger (2003) believe that organizational support is an upward commitment; once employees feel support from organizational support, it will strive to help organizations achieve their goals.

#### Teacher Self-Efficacy

Bandura first proposed self-efficacy concept, self-efficacy refers to the estimation and judgment of individuals who have the ability to complete a certain behavior (Bandura and Wood, 1989). Individuals with high self-efficacy are full of confidence, more investment in their work, will be regarded as challenges, dare to try and work hard. Based on the Social Cognitive Career Theory, individuals with higher professional self-efficacy firmly believe that efforts will have good results and tend to take positive measures to deal with the challenges in work.

Bandura (1986) believes that the formation and development of individual self-efficacy is mainly affected by four factors. First, past successive experience. This is also the most important factor affecting individual self-efficacy. The successful experience can help individuals produce strong self-efficacy. Second, indirect experience from others. If the individual sees people who have similar people have succeeded through their efforts, then they tend to believe that they also have the opportunity to succeed, and the self-efficacy is relatively high. Third, the evaluation of others and self-perceptions. When individuals have encouraged and perceived they are trusted, they will improve their self-efficacy. Fourth, emotional status and physiological status. Positive emotions helps the individual’s self-efficacy enhancement.

In the field of organizational behavior, the results of variables on self-efficacy are mainly focused on work performance and related behavior of work. (Sadri and Rovertson, 1993) use live research to explore the relationship between managers’ self-efficacy and their work performance. The results indicate that managers’ self-efficacy is an important variable that affects its management performance. The results of Lauschruger and Shamian (1994) show that the self-efficacy and its team’s work performance has a significant positive correlation. Many research conclusions have shown that self-efficacy is one of the most effective predictors of work performance, and there is a forward correlation between work performance, that is, employees with high self-efficacy generally produce higher work performance.

Teachers’ self-efficacy is the extension of self-efficacy. Ashton believes that teachers’ self-efficacy is a positive impact and help of teachers themselves (Ashton, 1984). Tschannen-Moren believes that teachers’ self-efficacy is the belief that teachers organize and perform specific teaching tasks in specific scenarios (Tschannen-Moren and Hoy, 2001). Hoover et al. (1987) believes that teachers’ self-efficacy is a kind of teaching ability and professional knowledge that teachers’ own teaching capabilities and expertise, and help students. Rianne et al. (2021) using relationship-focused reflection to improve teacher–child relationships and teachers’ student-specific self-efficacy. Trentham and Brogdon’s research shows that teachers’ self-efficacy is related to the attention of teachers’ work satisfaction and school leaders. On the basis of the four major information source research around Bandura’s self-efficacy, the teachers’ self-efficacy feels masterpiece experience, alternative experience, speech persuading and physiological psychological status (Trentham et al., 1985). At present, research on teachers’ self-efficacy still has a lot of rising space.

#### Work Engagement

The concept of working engagement originated from the research of Lodahl and Kejner (1965), researchers divided the work engagement into individuals in psychological identity and individuals who wish to meet their own self-esteem. Kahn (1990) believes that work engagement is a psychological state that employees integrate with self-behavior to achieve self-role and work role cognition, including three aspects: awareness of work, sensitivity in physiologically highly involved and sensitivity to self and others. Schaufeli et al. (2002) redefines the concept of work investment from the perspective of emotional and cognition, and believes that individuals are active, lasting emotional and motivation in the work, manifesting as vital, dedication, and focus on three levels. This study analyzes the viewpoint of Schaufeli et al. (2002) on work investment.

Work engagement is a positive psychological state that can play a positive impact in business management. The study found that variables of influential work engagement can be divided into three categories, namely, first, statistical characteristics of population. Studies have shown that individual factors such as age, gender, occupation, education, and marriage will affect work engagement (Schaufeli et al., 2016). (Schaufeli and Bakker, 2003) has pointed out that the age of employee work engagement is higher. Second, individual characteristics. Individual characteristics mainly include individual emotions, self-efficacy, and personality traits. Kahn (1990) is in the earliest state of psychological state to promote individual engagement, including significance, safety, and availability. The study of Xanthopoulou et al. (2009) shows that there is a stronger working engagement at a high level of self-efficacy. Third, work-related characteristics. According to the JD-R model, the work resource can effectively predict the work engagement. Sonnentag (2003) adopts empirical research to the work characteristics, organization support and work engagement have significant positive correlation (Sonnentag, 2003). Empirical research suggests that social support, supervision guidance, performance feedback, and job resources are available, and skill diversity can stimulate employee’s work engagement, resulting in higher performance (Saari et al., 2017). Many studies have shown that many factors in the organization have an impact on work engagement, including organizational support, interpersonal relationships, and equity affect employee’s work engagement.

### Hypothesis Development

#### Organizational Support and Teacher Self-Efficacy

Organizational support is the subjective feeling of employees to the organization, and will affect employees’ self-efficacy. Many studies have shown positive correlations between the two variables, the higher the organizational support, the stronger the self-efficacy. The organization supports high-quality employees will promote the improvement of self-efficacy by adopting active mentality dealing with difficulties and setbacks, reducing the negative and adverse effects of pressure. Cheng et al. (2020) confirmed that self-efficacy and organization support positive correlation. Caesens and Stinglhamber (2014) has shown that organizational support and self-efficacy have a positive effect on scientific research personnel performance and also confirmed that researchers have significant correlation between organizational support and self-efficacy. It can be seen that organizational support is positively correlation with self-efficacy.

Therefore, organizational support is an important way to enhance teachers’ self-efficacy and can predict the self-efficacy of preschool inclusive education teachers. Therefore, this study proposes the following hypotheses:

H1: Organizational support has a significant positive impact on teacher self-efficacy.

#### Organizational Support and Work Engagement.

The earliest OST by Eisenberger emphasized the two-way partnership between organizations and employees. If organizations are willing to give employees more support, care, and commitment, it will more motivate employees, enhance employees to stay in the organization, and contribute to the organization. Organizational support reflects the perception of employees on their attitudes and can positively affect employee attitudes, behavior, and performance (Eisenberger et al., 2002).

The impact of organizational support on the work engagement includes direct and indirect effects. Van den Broeck et al. (2014) indicates that the organization support has a positive role in working engagement. Caesens and Stinglhamber (2014) found that organizational support not only directly affects work engagement but also through self-efficacy that indirectly affects employee work engagement. Organizational support not only has a positive impact on work engagement (Lartey et al., 2021) but also be influenced by different mechanisms, such as self-efficacy, organizational identity, and organizational fairness (Du and Wang, 2021). Shantz et al. (2014) examined the commonly held assumption that a low level of work engagement leads to higher turnover intentions and employee deviant behavior. Therefore, this study proposes the following hypotheses:

H2: Organizational support has a significant positive impact on work engagement.

#### Teacher Self-Efficacy and Work Engagement

Self-efficacy is a self-excitation mechanism. People think that they have the ability to complete their own set goals and have a considerable effort and long-term adherence to overcome difficulties (Bandura, 2005). Studies have shown that self-efficacy will affect the inner motivation, work satisfaction, and work engagement (Federici and Skaalvik, 2011; Yakin and Erdil, 2012), and the higher the self-efficacy of teachers, the more it helps to wake up or maintain their own active working status. Researchers such as Xanthopoulou have also found that self-efficacy is an important antecedent variable affecting employee’s work engagement (Xanthopoulou et al., 2007). Self-efficacy may be an important determinant of work engagement (Llorens et al., 2007). Simbula et al. (2011) took Italian teachers as the research object and conducted three rounds of research on teachers’ self-efficacy and work engagement. The research shows that there is a significant correlation between teachers’ self-efficacy and work engagement.

This study predicts that preschool inclusive education teachers’ self-efficacy at work will have a positive impact on their work engagement. Thus, this study proposes the following hypotheses:

H3: Preschool inclusive education teachers’ self-efficacy has a significant positive impact on work engagement.H3: Preschool inclusive education teachers’ self-efficacy has a significant positive impact on work engagement.

#### The Mediating Role of Inclusive Educational Efficacy

How teachers’ sense of organizational support affects work engagement and what is its internal influence mechanism are the main problems that this study attempts to solve. According to the literature review, teachers’ self-efficacy is one of the mediating variables worthy of attention. Teachers’ self-efficacy refers to teachers’ judgment, belief, and feeling about the value of education and their ability to do a good job in education and actively affect children’s development (Llorens et al., 2007; Yakin and Erdil, 2012). Ouweneel et al. (2012) found that the change of self-efficacy was consistent with the change of students’ engagement. However, there is less in-depth analysis and demonstration of the influencing factors and mechanisms affecting employees’ work engagement, especially the lack of relevant research on the professional group of preschool inclusive education teachers. In fact, as a cognitive dynamic mechanism, self-efficacy is an individual’s belief in their own working ability, which is affected by individual characteristics and affects the individual’s working state. People with high self-efficacy tend to choose more challenging tasks and strive to achieve their goals through self-regulation. Although the antecedent variables in the above are different, the self-efficacy is in which a mediation effect is placed.

This study predicts that teachers’ self-efficacy plays a mediating role between teachers’ organizational support and work engagement in preschool inclusive education. Good organization supports the formation of work engagement and teachers’ self-efficacy in preschool inclusive education teachers. Moreover, teachers with high self-efficacy also have higher engagement to their work. Therefore, this study proposes the following hypotheses:

H4: Teacher self-efficacy has a mediating role in the relationship between organizational support and work engagement.

## Research Methodology

**FIGURE 1:**
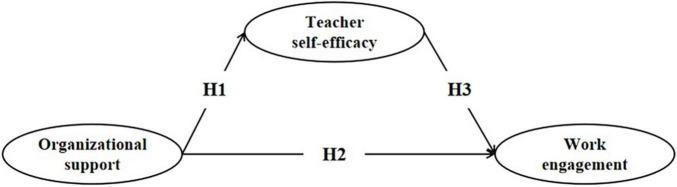
Research model.

This study examines the linkage between organizational support and work engagement in preschool inclusive education and the mediating role of preschool teachers’ inclusive educational efficacy. The conceptual model used in this study is presented in Figure 1.

### Instrument

To ensure the causal relationship between antecedents and work engagement, a pretest was conducted with participants drawn from the population for the main study. A total of 120 students (68% female) participated in return for course credit in a regular classroom setting. The results revealed self-efficacy and psychological capital with the merchant influence the work engagement. To ensure content validity, the items used to measure the constructs were adapted from the existing literature and modified to fit the study context. The measurement items for organizational support were adapted from Eisenberger et al. (2002). The measurement items for inclusive education self-efficacy were adapted from Sharma et al. (2012). The measurement items for work engagement were adapted from Rich et al. (2010) (as shown in Appendix Table 1).

As the original items were in English, we conducted a back-translation to ensure translation validity. First, a researcher whose native language is Chinese translated the source items from English into Chinese. Next, another researcher independently translated these items back into English. Subsequently, the two researchers compared the two English versions and jointly revised the first Chinese version of the items. Based on their feedback, minor modifications were made to improve the comprehensiveness and user-friendliness of the measurement items. A pretest of the survey instrument was conducted to conceptually validate the instrument. The final survey questionnaire is presented in Appendix Table 1. All items were measured on a 5-point Likert scale, ranging from 1 (not agree at all) to 5 (absolutely agree).

### Data Collection

The members of the thesis research group are university preschool education teachers, and many of the students taught have worked in kindergartens. In addition, the members of the thesis research group also have cooperative relations with many kindergarten teachers in their usual work. The subjects for the study were the kindergarten teachers in Suzhou, China. The survey was carried out through an online crowdsourcing platform in China, which provides functions equivalent to Amazon Mechanical Turk. The online survey platform used in this study is the most representative in China.

Data collection was conducted on January 2022. Participants were informed that their participation would assist in contributing to the development of integrated education as a result. The demographic characteristics of the final sample are summarized in Table 1. A total of 600 respondents were surveyed over a 4-week period. Finally, 568 responses were used for subsequent analyses after 32 incomplete and invalid responses were excluded. In terms of gender distribution, there are significantly more women (65.1%) than men (34.9), and the majority (57.6%) were public kindergarten. Further, 41.9% worked for less than 1 year, and 41.5% worked for 1–3 years.

**TABLE 1:** Demographics of the survey respondents (*N* = 568).

Demographics	Category	Frequency	%
Gender	Male	198	34.9
	Female	370	65.1
Kindergarten type	Public kindergarten	327	57.6
	Private kindergarten	241	42.4
Teaching time	≤1 year	238	41.9
	1–3 years	236	41.5
	3–6 years	56	9.9
	6–10 years	26	4.6
	≥10 years	12	2.1

## Data Analysis and Results

### Reliability and Validity

Construct reliability and validity were further examined through the confirmatory factor analysis (CFA). As shown in Table 2, the Cronbach’s α and composite reliability (CR) values for each construct ranged from 0.876 to 0.915, both of which were above the suggested threshold of 0.7 (Straub et al., 2004) and exhibited a satisfactory level of reliability. For construct validity, both convergent and discriminant validity were examined. Convergent validity was confirmed by examining the average variance extracted (AVE) and indicator loadings. As shown in Table 2, all AVE values were higher than the recommended level of 0.5 (Fornell and Larcker, 1981). The standard loadings of all items were above the desired threshold of 0.7 and significant at 0.001. This indicates good convergent validity (Chin et al., 1997).

**TABLE 2:** Results of confirmatory factor analysis.

Construct	Cronbach’s α	CR	AVE
Organizational support	0.876	0.849	0.504
Teacher self-efficacy	0.915	0.906	0.622
Work engagement	0.897	0.879	0.557

Discriminant validity was evaluated by comparing the square root of AVE and the correlation value. Discriminant validity was assessed by comparing the square root of AVE for each construct with the correlations between that construct and other constructs (Fornell and Larcker, 1981). According to Table 3, the square roots of the AVEs (diagonal elements) were larger than the interconstruct correlations depicted in the off-diagonal entries, suggesting adequate discriminant validity. Thus, discriminant validity was adequate.

**TABLE 3:** Results of discriminant validity testing.

	Mean	*SD*	OS	SE	WE
OS	4.342	2.023	***0.710***		
SE	4.773	1.942	0.430	***0.789***	
WE	3.973	1.893	0.429	0.572	***0.746***

*OS, organizational support; SE, teacher self-efficacy; WE, work engagement, Diagonal bold italics entries are square root of AVE; all others are correlations coefficients. M, mean, SD, standard deviation.*

### Hypothesis Testing

**FIGURE 2:**
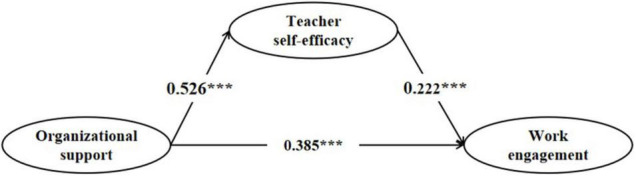
Results of the research model (***p < 0.001).

Figure 2 indicates that the nine hypothesized relationships are supported. Organizational support had a positive influence on preschool teachers’ self-efficacy (β = 0.526, *p* < 0.001). Organizational support and preschool teachers’ self-efficacy all had positive influences on work engagement (β = 0.385, *p* < 0.001; β = 0.222, *p* < 0.001), thus supporting H1, H2, and H3 (refer to Table 4).

**TABLE 4:** Hypotheses test.

Hypothesis path	Path coefficient	S.E	*t*-value	*p*-value	Results
H1: Organizational support → Teacher self-efficacy	0.526	0.053	9.863	***	Supported
H2: Organizational support → Work engagement	0.385	0.036	10.772	***	Supported
H3: Teacher self-efficacy → Work engagement	0.222	0.043	5.217	***	Supported

***p < 0.001.

Furthermore, to provide a more robust test of our results, control variables were included as direct antecedents of latent variables. According to the research results, gender, teaching time, and kindergarten type have no significant effect on the latent variables (*p* > 0.1). In future research, we will take the control variables as the independent variables to explore its impact on preschool teachers’ work engagement (refer to Figure 2).

Furthermore, we examined teacher self-efficacy mediation effect using the bootstrapping approach provided by Preacher and Hayes (2008). The use and test of the mediating effect is the main trend in management studies. According to Table 5, the indirect effect of teacher self-efficacy on the relationship between organizational support and work engagement is significant with a 95% bootstrap confidence interval, excluding zero. Preschool teachers’ self-efficacy plays a partial mediating role between organizational support and work engagement (β = 0.202, *p* < 0.001, CIs = [0.077, 0.305]). Organizational support not only directly affects teachers’ self-efficacy and work engagement in preschool inclusive education but also indirectly affects their work engagement through preschool teachers’ self-efficacy.

**TABLE 5:** Results of mediating effect analysis.

IV	M	DV	IV → M	IV → DV	M → DV	Indirect effect	CIs	Mediation
OS	SE	WE	0.526*** (0.053)	0.385*** (0.036)	0.222*** (0.043)	0.202*** (0.053)	[0.077, 0.305]	Yes
95% Bootstrap confidence intervals for indirect effect.								

*IV, independent variable; M, mediator variable; DV, dependent variable; CIs, confidence interval; OS, organizational support; SE, teacher self-efficacy; WE, work engagement. IV → DV is significant (M not included in the model); IV → M is significant; M → DV is significant (or the meaningful reduction in effect) of the relationships between the initial IV and DV in the presence of mediator.*

Significance at, ***p < 0.001; SE, Standard Errors in brackets.

## Discussion and Implications

### Discussion of Findings

This study yielded interesting findings. The results indicate that the organizational support significantly influences preschool teachers’ inclusive education self-efficacy and their work engagement. First, organizational support was found to have significant impacts on the preschool teachers’ self-efficacy. These findings are consistent with those of previous studies (Stankovic and Luthans, 1998; Chang et al., 2018), indicating that organizational support significantly impacts preschool teachers’ inclusive education self-efficacy.

Second, organizational support has a significant impact on work engagement. This suggests that organizational support can greater preschool teachers’ work engagement. Our findings extend those of previous studies (Karatepe and Aga, 2016; Ravindranath, 2017), suggesting that organizational support has a greater effect on work engagement.

Finally, this study confirms the mediating effect of teachers’ self-efficacy on the relationship between organizational support and work engagement. The mediating effect results of this study further verify the social exchange theory; preschool teachers’ self-efficacy plays a partial mediating role between organizational support and work engagement. The hypothesis is verified in line with the literature (Sharma et al., 2012; Monsen et al., 2013).

### Educational Contributions

This research has guiding significance for the cultivation of self-efficacy and work engagement of kindergarten teachers’ inclusive education in practice. Educational administrative departments and kindergarten leaders should fully understand and support teachers’ daily work and create a good organizational support environment.

First, adequate and effective organizational support should be provided. The education administrative department and kindergarten leaders should pool resources to provide sufficient and effective support for kindergarten teachers, so that teachers can feel the recognition and importance of their work units, as well as the humanistic care, material security, and professional leadership, prompting them to turn the support they feel into their work engagement to inclusive education.

Second, kindergarten teachers’ self-efficacy in inclusive education should be cultivated. Social cognitive theory shows that direct experience and alternative experience are important factors for individuals to form self-efficacy (Bandura, 1977). Therefore, in the context of developing preschool inclusive education, the education department should provide kindergarten teachers with a good platform for accumulating positive experiences in inclusive education.

Third, according to the research results, to promote the better development of inclusive education, kindergarten teachers are encouraged to actively participate in the work of inclusive education and to transform direct experience into professional strategies for follow-up work. In addition, kindergarten teachers should be provided with training, observation, and seminars oriented to the practical problems of inclusive education. Moreover, they should be guided through alternative learning and be able to master the theoretical and practical strategies of inclusive education.

### Limitations and Future Research

Based on the inclusive education theory, this study examines the impact of organizational support on inclusive education efficacy and work engagement. Moreover, the study examines the mediating role of inclusive educational efficacy between organizational support and work engagement. However, this research still has the following limitations. First, the research object is kindergarten teachers in Suzhou, China, and kindergarten teachers in other regions also need further research in future. Second, this study only explored the mediating effect of inclusive education efficacy, and other mediating effects also need to be studied in the future, such as identity.

## Data Availability Statement

The raw data supporting the conclusions of this article will be made available by the authors, without undue reservation.

## Ethics Statement

The studies involving human participants were reviewed and approved by the Normal School, Changshu Institute of Technology. Written informed consent for participation was not required for this study in accordance with the national legislation and the institutional requirements.

## Author Contributions

CJ designed the study and drafted the initial manuscript. CJ and JQ collected the data, performed statistical analysis, and drafted the initial manuscript. JQ and HL contributed to the revised manuscript. All authors discussed the results and contributed to the final manuscript.

## Conflict of Interest

The authors declare that the research was conducted in the absence of any commercial or financial relationships that could be construed as a potential conflict of interest.

## Publisher’s Note

All claims expressed in this article are solely those of the authors and do not necessarily represent those of their affiliated organizations, or those of the publisher, the editors and the reviewers. Any product that may be evaluated in this article, or claim that may be made by its manufacturer, is not guaranteed or endorsed by the publisher.

## Appendix

**APPENDIX TABLE 1:** Questionnaire items.

Construct	Measurement item	Reference
Organizational support (OS)	1. The organization attaches great importance to the objectives and values of the work done by employees.	Eisenberger et al., 2002
	2. In case of difficulties in work, the organization will help.	
	3. The organization will provide development opportunities for the future development of employees.	
	4. In work, the organization will consider the opinions put forward by employees.	
	5. The organization pays close attention to the living conditions of employees.	
	6. The organization will be proud of the achievements of its employees.	
Teacher self-efficacy (SE)	1. I firmly believe that I can achieve my ideals and goals.	Sharma et al., 2012
	2. I am confident that I can handle any situation freely.	
	3. No matter whether others object or not, I will get what I want.	
	4. I can keep calm in the face of difficulties.	
	5. I always have many ways to meet difficulties.	
	6. I am smart enough to face and solve any unexpected situation.	
Work engagement (WE)	1. I agree with the meaning of work.	Rich et al., 2010
	2. I often immerse myself in my work.	
	3. I am always full of passion in my work.	
	4. I always think clearly and feel happy after I put into work.	
	5. I can insist on completing the task when I encounter difficulties.	
	6. My work can inspire me to realize my own value.	
